# The interaction of gibberellin and melatonin promotes tobacco leaf growth and balances chemical components content in upper leaves

**DOI:** 10.3389/fpls.2026.1733182

**Published:** 2026-02-04

**Authors:** Haoran Xie, Yufan Yu, Shikang Yu, Mingxin Mu, Huizhan Gu, Dong Wang, Junju Li, Xiaoli Liu, Yifei Lu, Bi Ren, Liming Lu, Liqin Li

**Affiliations:** 1 Agronomy College, Sichuan Agricultural University, Chengdu, China; 2 Department of Tobacco Production Management, Sichuan Tobacco Company Guangyuan Branch, Guangyuan, China

**Keywords:** chemical component usability index, gibberellin, leaf, melatonin, tobacco

## Abstract

**Introduction:**

Tobacco (*Nicotiana tabacum* L.) upper leaves constitute approximately one-third of total leaf production, yet their smaller leaf area, compact structure, and imbalanced chemical composition limit suitability for cigarette manufacturing. This study investigated whether gibberellin (GA) and melatonin (MT) could promote upper leaf growth and balance chemical components.

**Methods:**

A two-factor randomized block design experiment was conducted to evaluate GA and MT effects on tobacco upper leaves. Morphological parameters, biomass accumulation, and chemical composition were measured. Structural equation modeling and TOPSIS (Technique for Order Preference by Similarity to Ideal Solution) analysis were employed to determine regulatory pathways and optimal hormone combinations.

**Results:**

Gibberellin significantly promoted upper leaf morphological development. The 75 mg/L GA treatment increased leaf width and leaf area by 12.96% and 17.69%, respectively, and significantly enhanced both fresh and dry weight (P<0.01). The GA-MT interaction significantly increased biomass accumulation (P<0.05). Structural equation modeling indicated GA primarily drove dry matter accumulation through direct regulation of leaf area (path coefficient, 0.47) and fresh weight (path coefficient, 0.39). Single hormone treatments improved chemical quality, with Chemical Component Usability Index (CCUI) ameliorated under 80 μmol/L MT and 75 mg/L GA treatments. TOPSIS analysis identified 75 mg/L GA + 80 μmol/L MT as the optimal combination (closeness coefficient, 0.644), effectively balancing biomass increase with optimized chemical coordination.

**Discussion:**

This study confirms gibberellin as the dominant factor regulating upper leaf morphology and biomass, while melatonin modulates chemical quality through interaction, providing a theoretical foundation and technical strategy for enhancing growth and chemical quality in tobacco upper leaves.

## 1 Introduction

Tobacco (*Nicotiana tabacum* L.) is an important economic crop cultivated worldwide (Zou et al., 2021). Typically, upper leaves refer to the top four to six leaves of tobacco plant, constituting approximately one-third of total tobacco leaf output. Upper tobacco leaves have high economic value, but due to factors such as weather and improper fertilization (Fan et al., 2013), they become unsuitable for ripening in the late growing season (Chen et al., 2019), Additionally, as they are positioned at the canopy top, upper leaves frequently face excessive light intensity, imbalanced carbon-nitrogen metabolism, and abnormal nicotine accumulation (Ma et al., 2025), thus making it more difficult to obtain high-quality tobacco leaves. The quality deficiencies of upper leaves primarily manifest in poor physical characteristics, inadequate leaf opening resulting in smaller leaf area, compact structure, excessive thickness, and imbalanced chemical composition (for instance, high nicotine content, low sugar-to-alkaloid ratio, etc) (Sun et al., 2018). Upper tobacco leaves account for 30–45% of total plant yield in China, yet their utilization rate in Class I and II cigarette blends remains below 15%—markedly lower than the 40% usage rate observed internationally (Hu et al., 2022). This substantial gap directly reflects quality limitations: high nicotine content (>3.5%) and excessive thickness restrict upper leaves to filler roles rather than flavor-contributing components in premium cigarette formulations, severely limiting their economic value realization. Thus, improving quality of upper tobacco leaves is important for enhancing tobacco output and farmer’s income.

To mitigate quality defects in upper tobacco leaves, current production primarily relies on cultural management practices, harvesting optimization, and curing process improvements. Moderately increasing planting density can enhance upper leaf maturity and oil content while improving the balance of chemical components (Huang et al., 2023; Chang et al., 2021). Fan et al. demonstrated that whole-stalk harvesting of upper leaves, compared with conventional leaf-by-leaf picking, facilitates nutrient translocation and yields more balanced chemical constituents along with superior aroma characteristics and economic returns (Fan et al., 2022). Fine-tuning curing protocols—specifically, shortening the steady-temperature duration at 36°C during the yellowing stage, elevating the wet-bulb temperature to 38°C, and maintaining relatively high wet-bulb conditions during the early 40°C phase—effectively reduces both the incidence and severity of ash hanging in upper leaves of the K326 cultivar, thereby increasing the proportion of high-grade leaf output (Liu et al., 2022). Nevertheless, these approaches exhibit delayed regulatory responses and require high technical expertise, constraining their practical adoption in large-scale production.

Plant hormones are small organic molecules produced endogenously in plant cells, playing important roles in plant growth and developmental as well as in response to various biotic and abiotic environmental stresses (Hirayama and Mochida, 2022). Plant growth regulators (PGRs) are artificially synthesized (or naturally extracted from microorganisms) chemical substances that have regulatory effects on plant growth and development, with chemical properties similar to plant hormones. Among them, gibberellins (GAs) directly affect upper leaf defects in tobacco: exogenous GA_3_ promotes mesophyll cell elongation, increasing leaf area to alleviate compact structure (Mi et al., 2025), while promoting dry matter accumulation by enhancing plant light capture efficiency and photosynthetic efficiency (Falcioni et al., 2018). Melatonin (MT) also plays an important role in improving crop quality; specifically, spraying EBR and MT in flue-cured tobacco can improve the sugar-to-alkaloid ratio, potassium and flavonoid compounds content, resulting in the improvement the quality of cured tobacco leaves (Wei et al., 2024). However, the synergistic interaction between GA and MT on upper leaf morphogenesis, tissue structure, and chemical coordination remains unelucidated, representing a critical knowledge gap for simultaneous yield and quality improvement.

Current research on GA and MT effects in tobacco has primarily concentrated on single-factor studies that emphasize morphological improvement (Mi et al., 2025) or chemical quality optimization (Wei et al., 2024). However, three critical knowledge gaps persist: (1) the synergistic mechanism by which GA-MT interaction influences upper leaf morphogenesis and tissue structure remodeling remains unclear; (2) the coordinated regulation of photosynthetic performance and carbon-nitrogen metabolism under combined hormone treatment has not been systematically investigated; and (3) empirical validation is lacking for the optimal concentration ratio to simultaneously enhance upper leaf biomass and coordinate chemical composition. This study addresses these gaps through a two-factor randomized block design that systematically evaluates GA-MT interaction effects on upper leaf growth, biomass accumulation, tissue structure, and chemical composition. Our objective is to elucidate the physiological mechanisms underlying hormonal synergy and identify a practical hormone combination strategy to resolve the problem of poor industrial utilization of upper leaves.

## 2 Materials and methods

### 2.1 Materials and experimental design

The tobacco variety used in this experiment was Yunyan 87, the main cultivated variety in Southwest China. This variety typically produces 18–20 effective leaves; in this experiment, topping was used to control the number of effective leaves to 18, with upper leaves defined as the top 6 leaves.

This experiment was conducted at the greenhouse of Sichuan Agricultural University, Chengdu Campus, Sichuan Province in 2022. The region has a subtropical humid monsoon climate with mean temperature of 28.4°C during the tobacco growth period (April–August). For pots experiment, experimental plastic pots with 40 cm in upper diameter, 25 cm in lower diameter and 35 cm in height were employed. Each pot contained 20 kg of soil with background nutrient concentrations of N 58 mg/kg, P_2_O_5_ 73 mg/kg, and K_2_O 108 mg/kg, supplemented with chemical fertilizer providing 3.50 g of pure N per pot at an N: P_2_O_5_: K_2_O ratio of 1:1.5:2. The used chemical fertilizers were nitrate phosphate fertilizer (32% pure nitrogen, 4% pure phosphorus), ammonium phosphate (11% pure nitrogen, 44% pure phosphorus), potassium sulfate (50% pure potassium) manufactured by Sichuan Jinye Fertilizer Manufacture Company.

A two-factor completely randomized trial design was employed in this experiment. Factor one was gibberellin (GA_3_) concentration with 4 levels (Level A1, 0 mg/L; Level A2, 25 mg/L; Level A3, 50 mg/L; Level A4, 75 mg/L). Factor two was melatonin concentration with 4 levels (Level B1, 0 μmol/L; Level B2, 40 μmol/L; Level B3, 80 μmol/L; Level B4, 120 μmol/L), resulting in a total of 16 treatments (**Table 1**). Each treatment had 10 replicates, and one tobacco seedling was transplanted in a single pot. The levels of gibberellin and melatonin were set according to our previous experiments (Chen et al., 2023).

**Table 1:** Experiment treatments.

Treatment	Gibberellin (mg/L)	Melatonin (μmol/L)
A1B1	0	0
A2B1	25	0
A3B1	50	0
A4B1	75	0
A1B2	0	40
A2B2	25	40
A3B2	50	40
A4B2	75	40
A1B3	0	80
A2B3	25	80
A3B3	50	80
A4B3	75	80
A1B4	0	120
A2B4	25	120
A3B4	50	120
A4B4	75	120

Tobacco seedlings were grown using the floating seedling technique. 65 days-old tobacco seedlings with similar size and shape were transplanted to the pots on April 23, 2022. Melatonin was sprayed at 50 DAT, coinciding with the rapid expansion phase of the 15th–18th leaves (upper leaf primordia). Gibberellin was sprayed at 71 DAT (1 day after topping), targeting the critical post-topping window when nutrient remobilization to upper leaves peaks. Each tobacco plant was sprayed with 50 ml of solution on both surfaces of the upper leaves until the mist condensed into droplets on the leaf surface without dripping.

### 2.2 Investigation of net photosynthetic rate of leaves

During the upper leaf harvesting period, the Li-6400 photosynthesis analyzer was used to measure the net photosynthetic rate (Pn) of each treated upper leaf. The measurement time was from 9:00am to 11:00am. The measurement position was the 3rd leaf from the top. When measuring, the instrument’s built-in light source was used and the light source intensity was set to 800 μ mol · m^-2^ s^-1^.

### 2.3 Leaf length and width measurement

For agronomic indicators measurement, five representative plants were selected, and upper leaf length, and upper leaf width were investigated according to the “Tobacco Agronomic Trait Survey and Measurement Method” (YC/T 142-2010) at 110 DAT (the upper leaf harvest stage). The measurement position was the 3rd leaf from the top.

Leaf area was calculated as:

Leaf Area = 0.6345 × Maximum Leaf Length × Maximum Leaf Width.

### 2.4 Upper leaf biomass determination

Upper leaf biomass was sampled and measured at 110 DAT. The top six leaves were collected from each sampled plant, and five tobacco plants were selected from each plot. Fresh weight (FW) was recorded immediately. Fresh tobacco leaves were then oven-killed at 105°C for 30 minutes and dried at 60-80°C to constant weight, and dry weight (DW) was recorded. The average single leaf weight (g/leaf) for the top six leaves was calculated.

### 2.5 Observation of leaf tissue structure

For tobacco leaf anatomical structure observation, conventional paraffin sectioning was performed. The third leaf from the top was selected, and tissue blocks (1 cm × 1 cm) were excised from the middle region, avoiding the main vein. Three biological replicates were prepared for each treatment. Sample fixation was carried out using FAA solution, followed by washing, dehydration, clearing, and paraffin embedding. Tissues were sectioned at a thickness of 11–13 µm using a rotary microtome. Sections were then deparaffinized, double-stained with safranin and fast green, cleared with xylene, and mounted with Canada balsam. For each tissue block, five slides were prepared. Microscopic observation and photography were conducted under a light microscope (Olympus BX53). For qualitative analysis, three fields of view were examined per slide; for quantitative measurements, five fields of view were analyzed per slide, ensuring the total number of fields met statistical requirements.

### 2.6 Tobacco leaf chemical composition analysis and CCUI calculation

Mature leaves were harvested and cured using the “Three-stage” curing process. After curing, tobacco leaves with B2F grade from each plot were selected for chemical composition analysis.

To do this, cured leaves were ground using a mill and passed through a 0.45 mm (40 mesh) sieve. The powdered samples were analyzed using a Futura/Proxima Continuous Flow Analyzer (French Alliance, France) following YC/T 159-2002 (Total sugars, Reducing sugar), YC/T 160-2002 (Nicotine), YC/T 161-2002 (Total nitrogen), YC/T 217-2007 (Potassium), and YC/T 162-2011 (Chlorine) — the standard methods for chemical composition determination in flue-cured tobacco. The nitrogen-to-alkaloid ratio (Total nitrogen/Nicotine), sugar-to-alkaloid ratio (Reducing Sugar/Nicotine), and potassium-to-chlorine ratio were then calculated.

Chemical Components Usability Index (CCUI) was employed to evaluate the chemical usability of flue-cured tobacco from each treatment (Jiang et al., 2017). The CCUI was calculated as follows.


$$\text{CCUI}=\sum_{j=1}^{m}W_{ij}\cdot C_{ij}$$

Where, W_ij_ is the weight assigned to each chemical indicator (**Table 2**), C_ij_ is the membership function value of the indicator; i=1, 2, …; j=1, 2, …. The function type and critical values were determined (**Table 3**). Two common types of membership functions were used as parabolic and S-shaped. Equation 1 represents the parabolic membership function, and Equation 2 represents the S-shaped membership function.

**Table 2:** Factor loading matrix, contribution rates, and variable weights.

Name	PC1	PC2	PC3	PC4	Composite score coefficient	Weight
Eigenvalue	4.316	1.991	1.181	1.015		
Variance explanation rate	47.95%	22.12%	13.12%	11.28%		
Total Alkaloids %	0.3402	0.3867	0.195	0.3453	0.3315	12.53%
Reducing Sugar %	0.3761	0.0126	0.4677	0.1358	0.275	10.40%
Total Sugar %	0.4124	0.2231	0.3387	0.0499	0.3145	11.89%
Total Nitrogen %	0.3130	0.4463	0.0007	0.1736	0.2842	10.75%
Potassium %	0.1325	0.3703	0.1660	0.7532	0.2670	10.09%
Chlorine %	0.2366	0.2764	0.6777	0.0890	0.2895	10.95%
Sugar/Alkaloid	0.469	0.0789	0.0879	0.1022	0.2809	10.62%
Nitrogen/Alkaloid	0.1799	0.611	0.2248	0.2247	0.2924	11.06%
Potassium/Chlorine	0.3859	0.0912	0.2889	0.4399	0.3099	11.72%

**Table 3:** Membership function types and inflection point values of chemical composition indicators of flue-cured tobacco.

Chemical indicator	Function type	x1	x3	x4	x2
Total Alkaloids	Parabolic	1.0	2.0	2.5	3.5
Reducing Sugar	Parabolic	10.0	19.0	25.0	30.0
Total Sugar	Parabolic	10.0	20.0	28.0	35.0
Total Nitrogen	Parabolic	1.1	1.8	2.0	3.0
Chlorine	Parabolic	0.1	0.3	0.5	1.0
Sugar/Alkaloid	Parabolic	3.0	8.0	13.0	18.0
Nitrogen/Alkaloid	Parabolic	0.2	0.6	1.0	1.5
Potassium	S-shaped	1.0			2.5
Potassium/Chlorine	S-shaped	2.7			10.0

(1) $$f(x)=\{\begin{array}{cc} 0.1x & x<x_{1},x>x_{2}\\ 0.9(x-x_{1})/(x_{3}-x_{1})+0.1 & x_{1}\leq x\leq x_{3}\\ 1.0 & x_{3}\leq x\leq x_{4}\\ 1.0-0.9(x-x_{4})/(x_{2}-x_{4}) & x_{4}<x\leq x_{2} \end{array}\;$$

(2) $$f(x)=\{\begin{array}{cc} 1.0 & x>x_{2}\\ 0.9(x-x_{1})/(x_{2}-x_{1})+0.1 & x_{1}\leq x\leq x_{\begin{array}{c} 2 \end{array}}\\ 0.1 & x<x_{1} \end{array}$$

Where, x_1_ is the lower critical value; x_2_ is the upper critical value; x_3_ is the lower limit of the optimal range; x_4_ is the upper limit of the optimal range, with x_2_ > x_4_ > x_3_ > x_1_.

### 2.7 Data processing

Data organization was performed using Microsoft Excel 2019. Two-way analysis of variance (ANOVA) was conducted using R version 4.5.0 (R Development Core Team). Duncan’s multiple range test (p < 0.05) for agronomic traits and biomass among the 16 treatments was performed using the agricolae package in R. Pearson correlation coefficients for agronomic traits and biomass were calculated using IBM SPSS Statistics 27. A piecewise structural equation model (SEM) for leaf biomass under different treatments was constructed using the lavaan package in R to explore the pathways and path coefficients of GA_3_ and MT concentration effects on tobacco biomass. The Technique for Order Preference by Similarity to Ideal Solution (TOPSIS) was performed using the plyr package in R to evaluate 16 treatments based on four benefit-type indicators: upper leaf area (m²), fresh weight (g), dry weight (g), and CCUI (unitless). Each indicator has equal weight (0.25). Normalization used vector method to eliminate scale effects. The positive ideal solution (PIS) consisted of the maximum value of each indicator across all treatments, while the negative ideal solution (NIS) comprised the minima. The closeness coefficient (Ci) was calculated as the relative distance to PIS. Graphs were generated using Origin 2025 and the ggplot2 package in R.

## 3 Results

### 3.1 The interaction of GA and MT significantly enhanced tobacco leaf photosynthetic rate (Pn)

**Figure 1:**
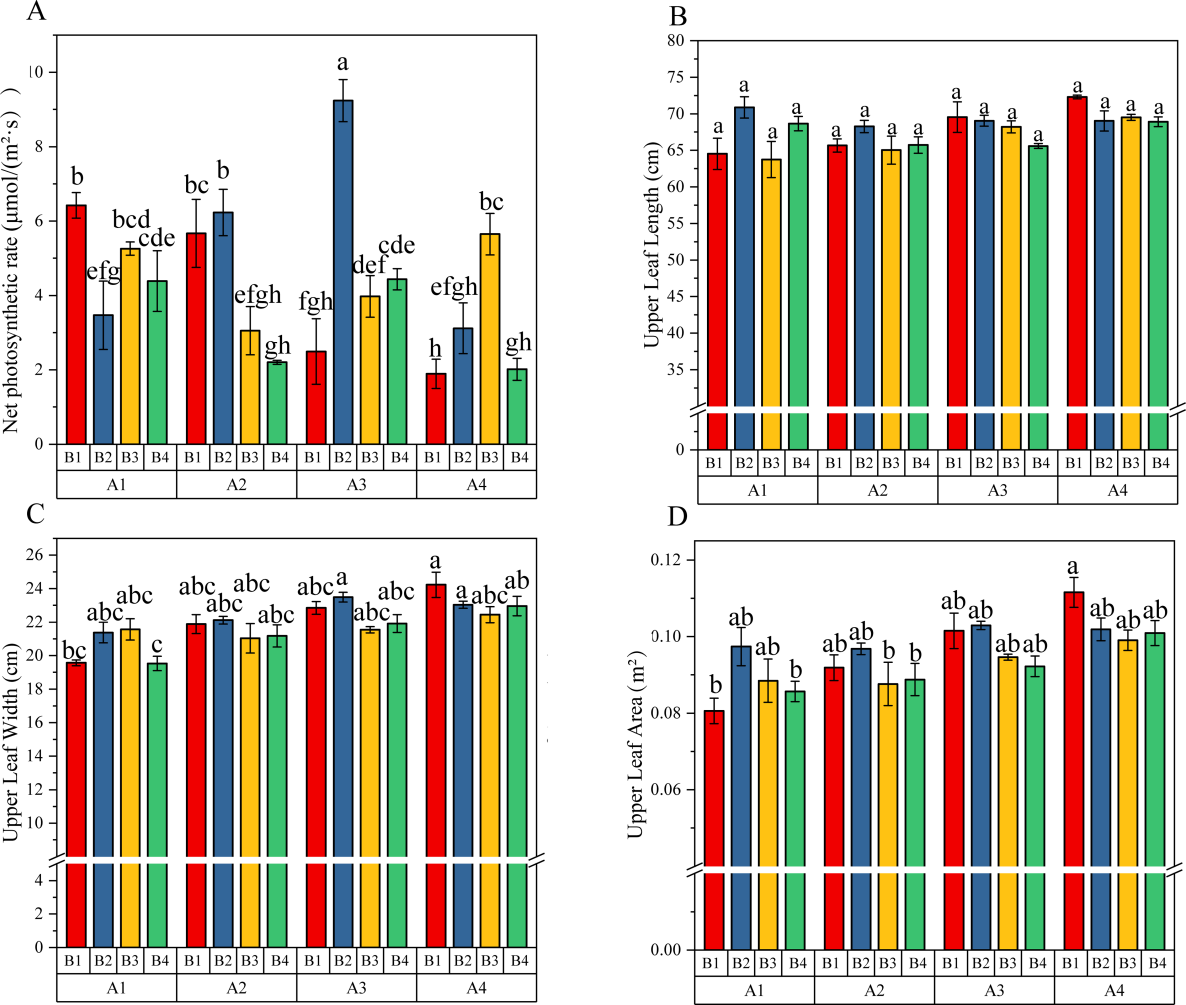
Interactive effects of GA₃ and MT on **(A)** leaf net photosynthetic rate, **(B)** leaf length, **(C)** leaf width, and **(D)** leaf area of upper leaves. Values are mean ± SD (n=3). Different lowercase letters indicate significant differences among treatments (*P* < 0.05).

During the harvesting period, the net photosynthetic rate of upper tobacco leaves in each treatment was measured. The results showed that both GA_3_ and MT application could significantly affect the photosynthetic characteristics of leaves, and there was a highly significant interaction between the two treatments (**Table 4**). In MT treatment, the net photosynthetic rate was highest at a concentration of 40 μmol/L and significantly higher than the other two high concentration treatments; In GA_3_ treatment, the net photosynthetic rate was highest at 50mg/L and significantly higher than the other three treatments. Among all the treatments of GA_3_ and MT interaction, A3B2 treatment had the highest net photosynthetic rate, while A4B1 treatment had the lowest (**Figure 1**).

**Table 4:** The variance analysis of the effects of GA_3_ and MT interaction on net photosynthetic rate, leaf area, and biomass of upper leaves.

	Treatment	*F*	*P*
Net photosynthetic rate	Gibberellin	13.3308	8.00E-06***
	Melatonin	16.1726	1.00E-06***
	Gibberellin × Melatonin	20.9405	5.15E-11***
Upper leaf length	Gibberellin	1.6165	0.2049
	Melatonin	0.8119	0.4967
	Gibberellin × Melatonin	0.6984	0.7054
Upper leaf width	Gibberellin	5.2759	0.0045**
	Melatonin	0.9747	0.4168
	Gibberellin × Melatonin	0.6274	0.7650
Upper leaf area	Gibberellin	3.8318	0.0188*
	Melatonin	1.1107	0.3592
	Gibberellin × Melatonin	0.4835	0.8747
Fresh weight	Gibberellin	7.5823	0.0006***
	Melatonin	1.3620	0.2720
	Gibberellin × Melatonin	2.4700	0.0288*
Dry weight	Gibberellin	8.0116	0.0004***
	Melatonin	0.8895	0.4571
	Gibberellin × Melatonin	2.6630	0.0198*

* Indicates significant difference at *P* ≤ 0.05; ** Indicates *P* ≤ 0.01, highly significant difference; *** Indicates *P* ≤ 0.001, extremely significant difference.

### 3.2 Gibberellin significantly improved tobacco leaf development

To investigate whether the interaction of gibberellin (GA) and melatonin (MT) affects the growth of flue-cured tobacco plants, we measured plant height and stem circumference at the topping stage, as well as upper leaf length and width at the upper leaf harvest stage. As shown the two-way ANOVA results in **Table 4**, GA_3_ significantly regulated tobacco leaf morphology. GA_3_ application significantly increased leaf width (*F* = 5.2759, *P* = 0.0045) and leaf area (*F* = 3.8318, *P* = 0.0188). Leaf width showed an increasing trend with GA_3_ concentration increased, reaching its maximum average value of 23.17 cm under the A4 treatment (75 mg/L GA_3_), representing a 12.96% increase compared to no GA_3_ application. This increase in leaf width contributed to greater upper leaf area, which peaked at 0.10 m² under the A4 treatment, a 17.69% increase over the no- GA_3_control. In contrast, melatonin had no significant effect on any agronomic traits (plant height, stem circumference, leaf length, leaf width and leaf area, *P* > 0.05). Furthermore, the GA_3_ × MT interaction was not statistically significant for any trait (*P* > 0.05). Multiple comparisons in **Figure 1** further confirmed that GA_3_ is the key factor inducing changes in tobacco leaf morphology and promotes upper leaf expansion.

### 3.3 Gibberellin and melatonin interaction significantly increased tobacco upper leaf biomass

**Figure 2:**
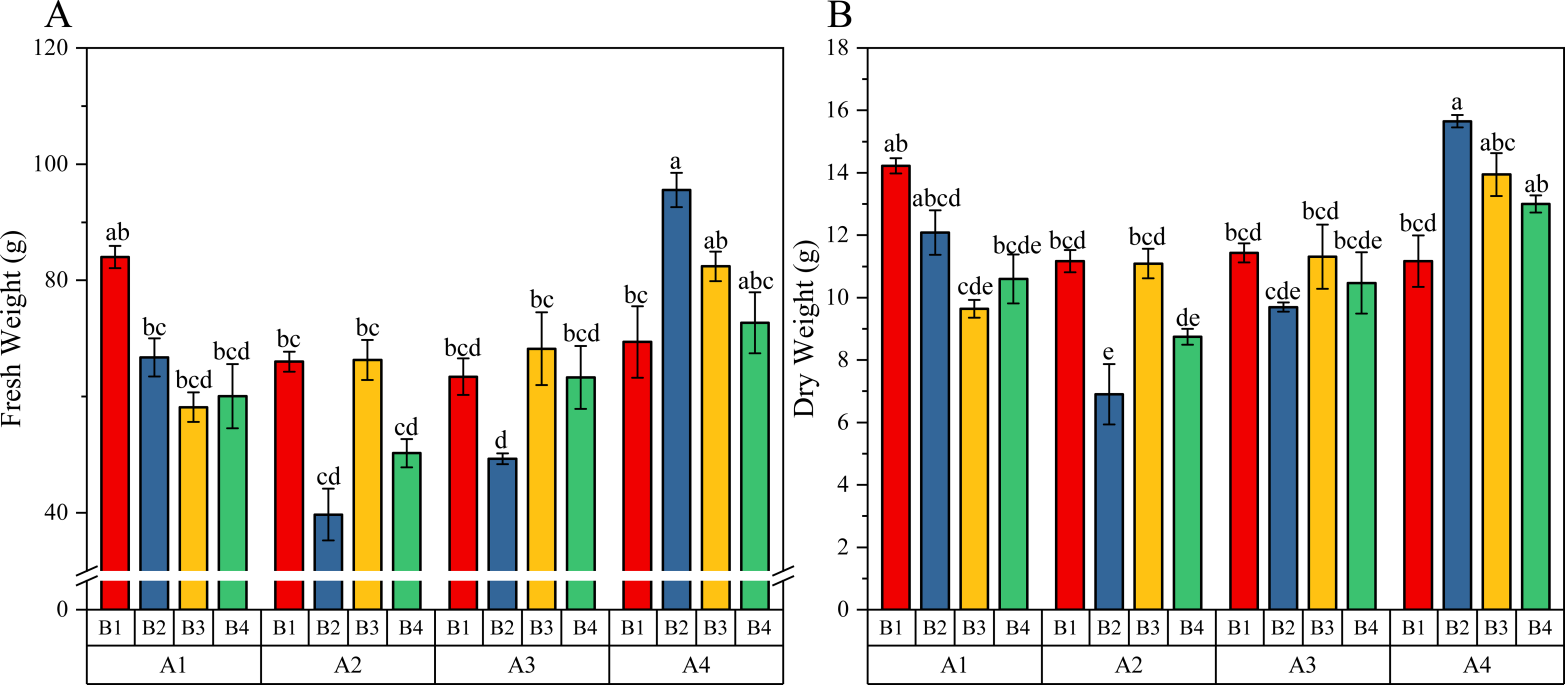
Effects of gibberellin and melatonin on **(A)** fresh weight and **(B)** dry weight of upper leaves of tobacco. Values are mean ± SD (n=3). Different lowercase letters indicate significant differences among treatments (*P* < 0.05).

Two-way ANOVA results (**Table 4**) showed that GA_3_ significantly increased the fresh weight (*F* = 7.5823, *P* = 0.0006) and dry weight (*F* = 8.0116, *P* = 0.0004) of tobacco upper leaves. The highest fresh weight (80.02 g) and dry weight (13.44 g) were achieved under 75 mg/L GA_3_ (A4) treatment. While the lowest fresh weight (55.56 g) and dry weight (9.48 g) observed under 25 mg/L GA_3_ treatment. In contrast, MT application had no significant effect on either fresh weight (*P* = 0.2720) or dry weight (*P* = 0.4571). Notably, the GA × MT interaction had a significant effect on both fresh weight (*F* = 2.4700, *P* = 0.0288) and dry weight (*F* = 2.6630, *P* = 0.0198), indicating synergistic regulation of biomass accumulation. Upper leaf fresh weight and dry weight were the highest under the A4B2 treatment (75 mg/L GA_3_ + 40 μmol/L MT), while fresh weight was the lowest under A3B2 (50 mg/L GA_3_ + 80 μmol/L MT), and dry matter accumulation was the lowest under A2B2 (25 mg/L GA_3_ + 40 μmol/L MT) (**Figure 2**).

### 3.4 Path analysis of the impact of GA and MT interaction on upper leaf biomass

**Figure 3:**
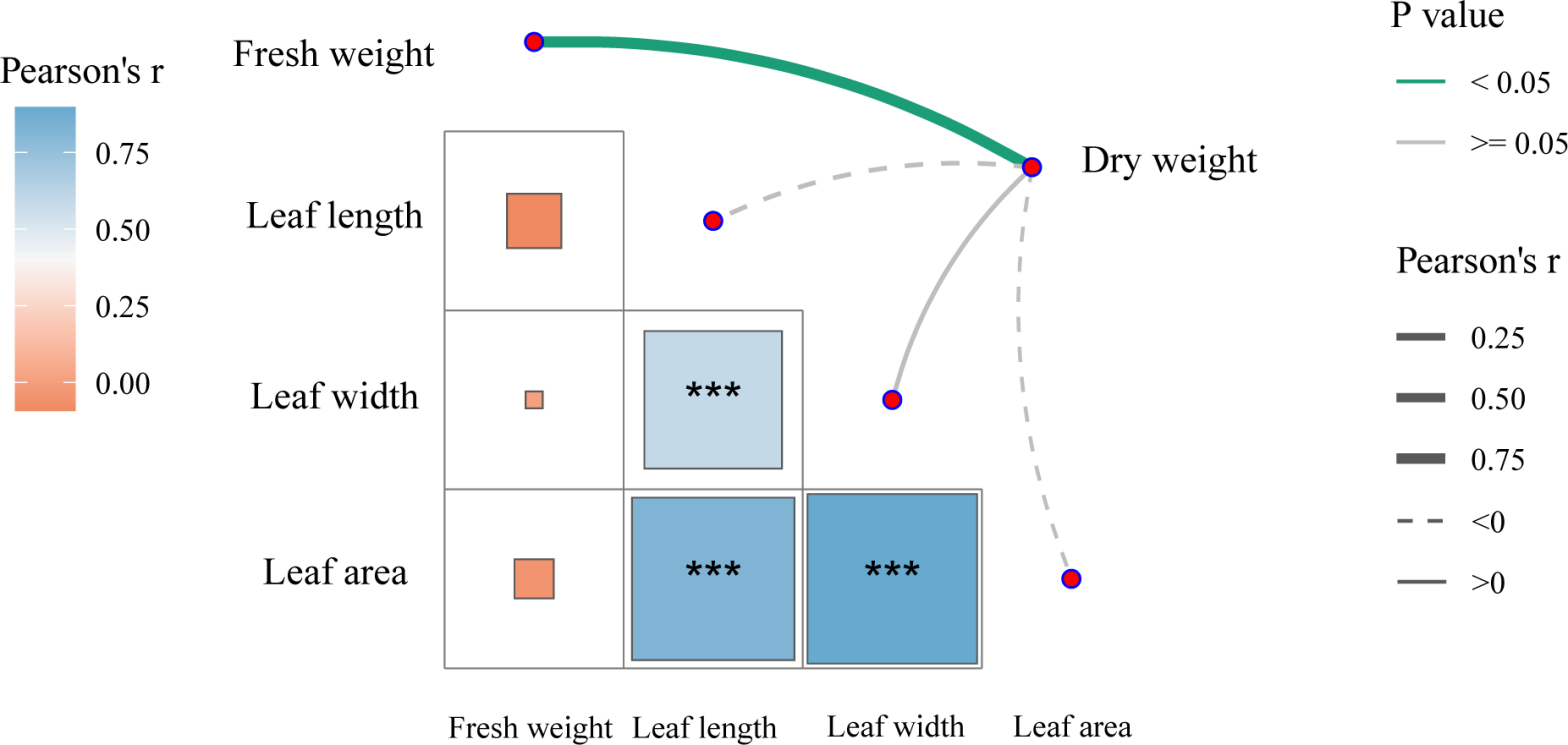
Pearson correlation analysis between the biomass of upper leaves and agronomic traits of tobacco plants. *** indicates significance at the level of *P* < 0.001.

Pearson correlation analysis (**Figure 3**) showed that upper leaf length, width, and area were all highly significantly positively correlated with each other (*P* < 0.01). Fresh weight and dry weight of upper leaves showed a significant positive correlation (*P* < 0.05).

**Figure 4:**
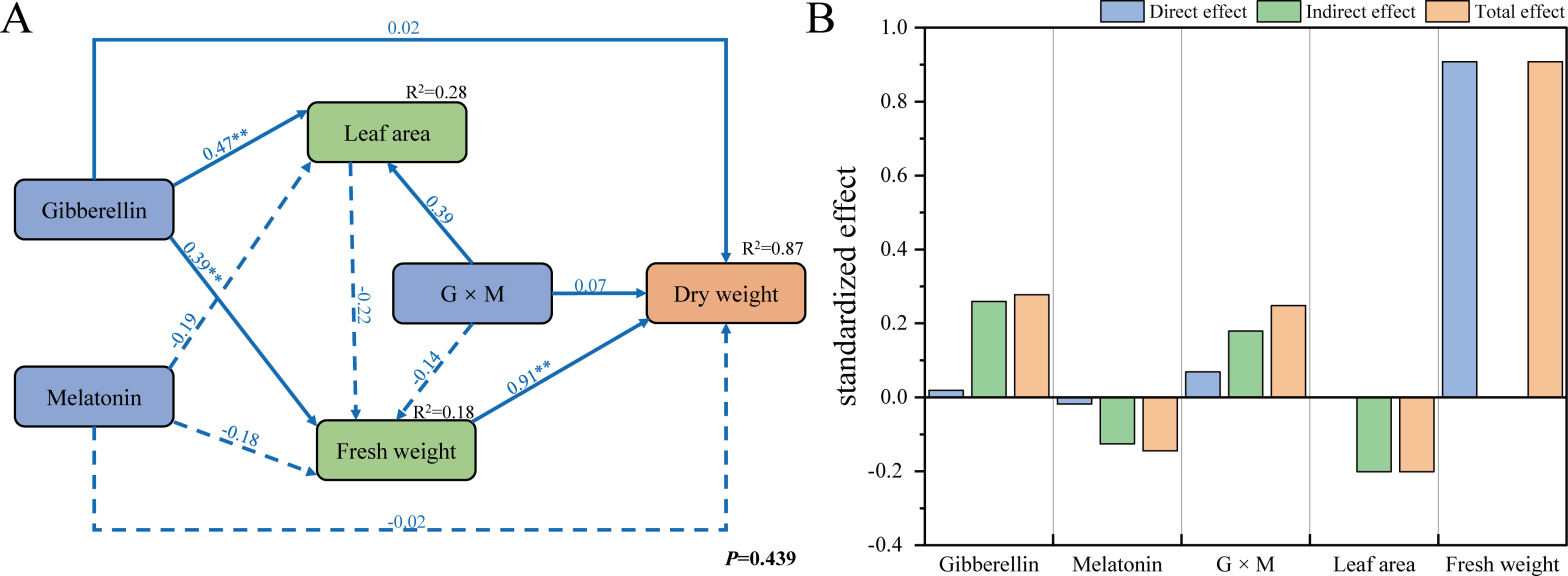
Path analysis of the effects of gibberellin and melatonin interaction on the biomass of upper leaves of tobacco using a piecewise structural equation model **(A)** and the standardized effect values of each factor **(B)**. Solid and dashed arrows in the figure represent significant positive and negative path relationships, respectively. The values are standardized path coefficients. ** indicates significance at the 0.01 level.

To explore how GA_3_, MT, and their interaction affect biomass, a piecewise structural equation model (SEM) was constructed for leaf area and biomass. As shown in **Figure 4A**, the model fit was good (*P* = 0.439). GA_3_ had a significant (*P* < 0.05) direct effect on leaf area and fresh weight, with path coefficients of 0.47 and 0.39, respectively. The relationship between leaf area and fresh weight itself was not significant. Increased fresh weight ultimately led to dry matter accumulation in the upper leaves. Calculation of standardized effect values (**Figure 4B**) showed that fresh weight had the highest direct effect on dry weight (0.908). By calculating the total effects of GA_3_, MT, and their interaction, GA_3_ and the GA_3_ × MT interaction promoted dry weight accumulation in upper leaves, with total effects of 0.278 and 0.248, respectively. The total effect of MT on upper leaf dry weight was -0.145.

### 3.5 The influence of GA and MT interaction on the tissue structure of upper leaves

**Figure 5:**
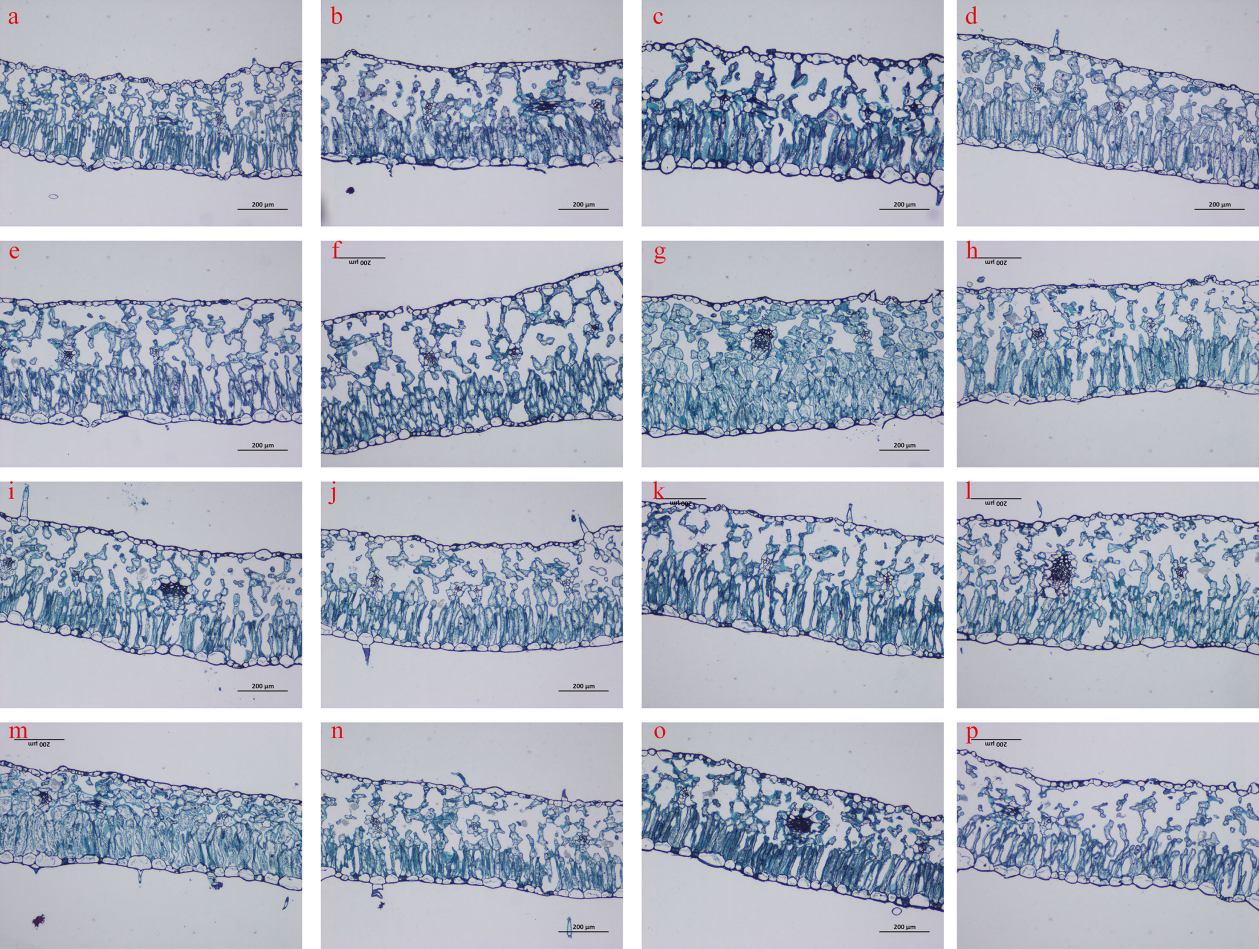
Interactive effects of GA_3_ and MT on the leaf structure of upper tobacco leaves. Panels **(a–p)** in **Figure 5** correspond to the 16 treatments A1B1, A1B2, A1B3, A1B4, A2B1, A2B2, A2B3, A2B4, A3B1, A3B2, A3B3, A3B4, A4B1, A4B2, A4B3, A4B4; see **Table 1** for details.

The tissue structure of tobacco leaves treated differently was observed, and the results are shown in **Figure 5**. The effect of GA_3_ and MT treatments on tobacco leaf development varied, exhibiting a certain dose effect. As the spraying concentration increased, the sponge tissue of the leaves showed a change of “dense-sparse-dense”. Meanwhile, the interaction between GA_3_ and MT had a positive promoting effect on the tissue structure of leaves. Among them, the tissue structure treated with A4B1 (**Figure 5m**) was the most compact, while the leaf tissue structure treated with A4B4 (**Figure 5p**) was the most loose. It can be also observed that the tissue structure of leaves treated with GA_3–_50 mg/L (A1B2, A2B2, A3B2, A4B2) and MT 40 μ mol/L (A3B1, A3B2, A3B3, A3B4) is relatively loose.

### 3.6 Gibberellin and melatonin significantly improve tobacco upper leaf chemical quality

Total plant alkaloids (TPA), reducing sugar (RS), total sugar (TS), total nitrogen (TN), potassium (K) and chlorine (Cl) are important chemical components and indicators for evaluating tobacco leaf quality. To some extent, The Chemical Components Usability Index (CCUI) of tobacco leaf represents the coordination and industrial availability of the chemical composition of tobacco leaves. In our study, the chemical composition of B2F tobacco leaves treated with different treatments was determined, and their CCUI values were calculated (**Table 5**). The result showed that the CCUI values of each treatment were distributed between 74.70-39.26. Different treatments had a significant impact on the chemical composition of B2F tobacco leaves, and the concentrations of GA_3_ and MT were the key factors affecting CCUI.

**Table 5:** Chemical composition indicators and CCUI of tobacco leaves from different treatments.

Treat-ment	TPA (%)	RS (%)	TS (%)	TN (%)	K (%)	Cl (%)	TS/A	N/A	K/Cl	CCUI
A1B3	3.01 ± 0.22ef	16.12 ± 0.17ef	19.70 ± 0.01a	2.55 ± 0.20bc	2.30 ± 0.02b	0.16 ± 0.02b	6.54 ± 0.00a	0.85 ± 0.00c	14.38 ± 10.97a	74.70
A4B1	2.81 ± 0.31f	14.41 ± 0.17f	16.70 ± 0.20bc	2.80 ± 0.21abc	2.27 ± 0.03a	0.30 ± 0.03a	5.95 ± 0.03b	1.00 ± 0.03a	7.57 ± 1.23bc	71.62
A2B3	3.63 ± 0.22cd	13.64 ± 0.36cd	17.02 ± 0.22b	2.45 ± 0.17c	1.76 ± 0.03c	0.18 ± 0.03c	4.69 ± 0.00c	0.67 ± 0.00h	9.78 ± 5.54b	58.50
A2B4	3.35 ± 0.25de	14.95 ± 0.17de	16.21 ± 0.21bc	2.61 ± 0.39abc	1.27 ± 0.03a	0.36 ± 0.03a	4.84 ± 0.05c	0.78 ± 0.05e	3.53 ± 0.69c	53.36
A4B3	3.18 ± 0.02e	11.50 ± 0.36e	13.12 ± 0.11h	2.81 ± 0.23abc	2.09 ± 0.02a	0.42 ± 0.02a	4.12 ± 0.05de	0.88 ± 0.05b	4.98 ± 0.84bc	52.17
A1B4	3.76 ± 0.21bc	11.60 ± 0.34bc	16.34 ± 0.38bc	2.56 ± 0.27bc	1.52 ± 0.03b	0.34 ± 0.03b	4.35 ± 0.03d	0.68 ± 0.03h	4.47 ± 0.04c	50.74
A2B1	3.55 ± 0.14c	14.66 ± 0.36c	16.76 ± 0.16bc	2.52 ± 0.31c	1.17 ± 0.01c	0.23 ± 0.01c	4.73 ± 0.05c	0.71 ± 0.05g	5.09 ± 1.96bc	50.69
A2B2	3.64 ± 0.38cd	13.14 ± 0.14cd	14.94 ± 0.28ef	2.75 ± 0.16abc	2.21 ± 0.01a	0.57 ± 0.01a	4.09 ± 0.03e	0.76 ± 0.03f	3.88 ± 0.08c	50.43
A3B2	3.06 ± 0.13ef	11.53 ± 0.36ef	13.33 ± 0.14gh	2.71 ± 0.29abc	1.37 ± 0.04a	0.29 ± 0.04a	4.36 ± 0.05d	0.89 ± 0.05b	4.72 ± 0.57bc	49.92
A3B3	3.64 ± 0.37cd	11.40 ± 0.32cd	12.44 ± 0.08h	2.93 ± 0.17ab	2.13 ± 0.03a	0.37 ± 0.03a	3.41 ± 0.03g	0.80 ± 0.03de	5.76 ± 1.01bc	46.56
A1B1	3.79 ± 0.13bc	14.12 ± 0.34bc	15.92 ± 0.88cd	2.81 ± 0.68abc	2.39 ± 0.07a	0.97 ± 0.07a	4.20 ± 0.13de	0.74 ± 0.13f	2.46 ± 0.01c	43.67
A4B2	3.56 ± 0.14c	11.21 ± 0.14c	13.11 ± 0.15h	2.84 ± 0.02abc	1.81 ± 0.01a	0.51 ± 0.01a	3.68 ± 0.02f	0.80 ± 0.02d	3.55 ± 0.16c	43.16
A1B2	4.30 ± 0.34a	10.22 ± 0.36a	14.23 ± 0.32f	2.78 ± 0.40abc	1.56 ± 0.02a	0.37 ± 0.02a	3.3 ± 0.03h	0.65 ± 0.03i	4.22 ± 0.81c	42.80
A4B4	3.69 ± 0.17bcd	14.53 ± 0.30bcd	15.16 ± 0.03de	2.96 ± 0.01a	1.52 ± 0.03a	0.67 ± 0.03a	4.09 ± 0.03e	0.80 ± 0.03d	2.27 ± 0.22c	42.22
A3B1	3.31 ± 0.32de	12.34 ± 0.03de	14.16 ± 0.39fg	2.75 ± 0.13abc	1.32 ± 0.01a	0.80 ± 0.01a	4.26 ± 0.03de	0.83 ± 0.03c	1.65 ± 0.03c	39.58
A3B4	4.05 ± 0.12ab	10.81 ± 0.33ab	12.52 ± 0.11h	2.99 ± 0.17a	1.57 ± 0.02a	0.37 ± 0.02a	3.09 ± 0.02h	0.74 ± 0.02f	4.24 ± 0.25c	39.26

TPA, Total Plant Alkaloids; RS, Reducing Sugar; TS, Total Sugar; TN, Total Nitrogen; K, Potassium; Cl, Chlorine; TS/A, Total Sugar/Alkaloid Ratio; N/A, Nitrogen/Alkaloid Ratio; K/Cl, Potassium/Chlorine Ratio; CCUI, Chemical Components Usability Index. Different lowercase letters indicate significant differences among treatments (*P* < 0.05).

When a single high concentration hormone was applied, the industrial usability of the upper leaves showed excellent performance. The industrial availability of A1B3 treatment (0 mg/L GA_3_ +80 μmol/L MT) was the highest (CCUI = 74.70), followed by A4B1 treatment (75 mg/L GA_3_ +0 μmol/L MT) (CCUI = 71.62). The tobacco leaves treated with A1B3 showed good performance in reducing sugar, total sugar, sugar alkali ratio, potassium content, potassium chloride ratio and other indicators, and the chemical composition of the tobacco was relatively coordinated. However, when treated with a combination of GA_3_ and MT at higher concentrations, the CCUI value of the upper tobacco leaves sharply decreased, among them the CCUI values of A3B4 (CCUI = 39.26) and A4B4 (CCUI = 42.22) were at the lowest level. This result indicates that exogenous hormone application is necessary to optimize tobacco leaf chemical quality.

### 3.7 Comprehensive evaluation of different gibberellin and melatonin concentration treatments

**Figure 6:**
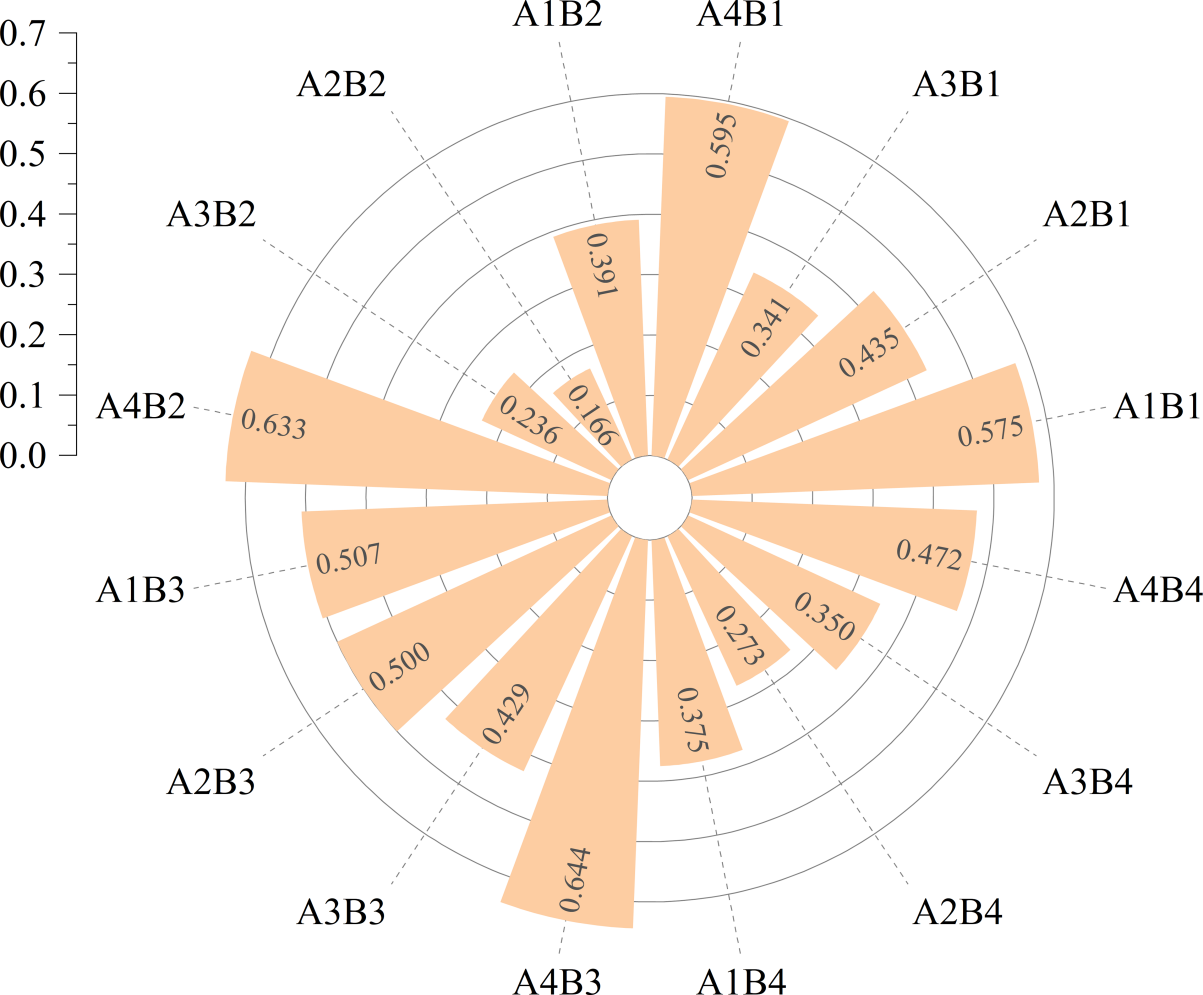
TOPSIS evaluation of GA-MT treatments based on four equally-weighted indicators (area, fresh weight, dry weight, CCUI). Data were normalized using vector method. A4B3 (75 mg/L GA_3_ + 80 μmol/L MT) achieved the highest closeness coefficient (Ci = 0.644).

TOPSIS (The Technique for Order Preference by Similarity to an Ideal Solution) analysis was carried out to comprehensively evaluate the effects of different GA_3_ and MT treatments on upper leaf area, upper leaf fresh weight, dry weight, and CCUI values of tobacco upper leaves. The results showed that the A4B3 treatment (75 mg/L GA_3_ + 80 μmol/L MT) had the highest closeness coefficient (0.644) (**Figure 6**), suggesting that applying 75 mg/L GA_3_ + 80 μmol/L MT not only increases upper leaf biomass, promotes leaf expansion but also enhances upper leaf quality.

## 4 Discussion

### 4.1 Both GA and MT can promote photosynthesis in tobacco leaves

GA is an important plant endogenous hormone that can promote plant growth and also have a certain enhancing effect on photosynthesis. For example, GA application improved photosynthetic capacity and biomass in wheat under zinc oxide (ZnO) stress (Iftikhar et al., 2019). Soaking pea seeds in GA_3_ solution enhanced antioxidant capacity and photosynthetic capacity under salt stress (Guo et al., 2022). Spraying 10 μM GA_3_ and 100 mg/kg S (elemental sulphur) enhanced photosynthetic capacity and sulfur assimilation in shepherd’s purse under cadmium stress (Masood et al., 2016). Elevated levels of bioactive GA_4_ in tobacco enhanced biomass and photosynthetic efficiency (Moreno et al., 2016). In studies on *Camellia oleifera*, it has been shown that GA treatment can increase the net photosynthetic rate of the first and sixth leaves. After application of 100 mg/L GA, the net photosynthetic rates of the first and sixth leaves increased by 59.55% and 44.90% (Wen et al., 2018).

MT plays a widespread role in plant stress responses; by boosting photosynthesis it helps plants cope with adverse conditions. Exogenous MT preserves high photosynthetic capacity in wheat and thereby alleviates flooding injury. (Ma et al., 2022). Under simulated drought stress, foliar melatonin application alleviates the PEG-6000-induced restriction on soybean (*Glycine max (L.) Merrill*) photosystem II (PSII), resulting in increased leaf area index and greater dry-matter accumulation (Zhang et al., 2019). Foliar application of 50 μM melatonin on tobacco alleviates the photosynthetic inhibition caused by high-nitrate stress (Hafeez et al., 2025).

Our study obtained similar results. In terms of net photosynthetic rate (Pn) of leaves, neither GA_3_ nor MT applied alone showed a significant increase over the control; however, a highly significant interaction between the two factors was detected (**Table 4**). The combination of 50 mg/L GA_3_ + 40 μmol/L MT produced the highest Pn, which was significantly greater than that of the control and all other treatments. This indicates that the interaction between GA_3_ and MT markedly improves the photosynthetic performance of tobacco leaves, laying the foundation for increased leaf biomass.

The significant interaction between GA_3_ and MT on Pn suggests a synergistic regulatory mechanism rather than a simple additive effect. GA_3_ primarily promotes leaf expansion and mesophyll cell elongation, thereby increasing the structural capacity for photosynthesis. In contrast, MT perhaps enhances photosynthetic efficiency by stabilizing photosystem II and alleviating oxidative stress. When applied together, GA_3_ provides an enlarged photosynthetic surface, while MT maintains the functional integrity of the photosynthetic apparatus, resulting in a pronounced increase in Pn.

### 4.2 Gibberellin is the dominant factor regulating morphology and biomass of tobacco upper leaves

Plants leaves size is determined by both cell division and expansion (Gonzalez et al., 2012). Gibberellin (GA) plays a crucial role in the leaf cell expansion process. In maize, GA enhances the polar elongation of cells in the leaf growth zone, thereby influencing the final leaf morphology (Sprangers et al., 2020). In another study, GA application can alleviate the reduced leaf elongation rates caused by salt and osmotic stress by upregulating genes associated with cell expansion (Xu et al., 2016). In our study, GA_3_ emerged as the core factor regulating the morphology of tobacco upper leaves. Spraying GA_3_ at the topping stage significantly promoted the increase in upper leaf width and leaf area. As GA_3_ concentration increased, this effect also escalated. This result aligns with the classical theory that gibberellin promotes cell elongation, thereby increasing leaf area (Jones and Kaufman, 1983).

Furthermore, our result also showed that GA_3_ significantly increased the biomass of upper leaves. Under 75 mg/L GA_3_ treatment, dry matter accumulation in upper leaves peaked at 13.44 g, increased by 15.53% compared to control. Structural equation modeling further revealed that GA_3_ drives dry matter accumulation primarily through direct promotion of leaf area (path coefficient, 0.47) and fresh weight (path coefficient, 0.39). This enhancement in dry matter accumulation may be linked to the fact that GA_3_ can increase photosynthetic leaf area or improve photosynthetic efficiency. Previous studies support this hypothesis as described in 4.1 section.

However, it must be noted that dry matter accumulation is not the primary factor contributing to the poor usability of upper leaves. In fact, excessive dry matter often leads to overly thick and “heavy” leaves, which reduces their industrial value. Our results imply that GA_3_ improves usability not just by increasing mass, but by “stretching” the leaf structure and expanding the leaf area, which prevents the excessive physical thickening in upper leaves.

### 4.3 Combined application of GA and MT alters the chemical components of tobacco leaves

Different chemical compound levels and compositions in tobacco leaf leads to variations in sensory quality and style in tobacco products (Hao et al., 2024). In our study, the combined application patterns of gibberellin (GA) and melatonin (MT) significantly altered the chemical coordination of upper tobacco leaves, directly impacting their CCUI. Our results showed that single hormones at appropriate concentrations effectively enhanced leaf quality. MT alone (80 μmol/L, A1B3) achieved the highest CCUI of 74.70, with appropriate levels of reducing sugar (16.1%), total sugar (19.7%), and sugar-to-alkaloid ratio (6.54). This may stem from MT’s promotion of carbon assimilation (Teng et al., 2022) and regulation of ion balance (Sheikhalipour et al., 2022). While GA_3_ alone (75 mg/L, A4B1, CCUI, 71.62) achieved quality improvement by maintaining lower total plant alkaloids (2.81%) and reasonable potassium content (2.27%). However, when both hormones were applied at high concentrations (e.g., A3B4, A4B4), CCUI values plummeted to 39.26–42.22, exhibited abnormal accumulation of total plant alkaloids accompanied by a sharp decrease in sugar content (Total Sugar), leading to severe imbalance in the sugar-to-alkaloid ratio (TS/A), coupled with a decline in the potassium-to-chlorine ratio (K/Cl), suggesting a significant antagonistic effect on upper leaf quality. This antagonism effect likely originates from the competition in hormone signaling pathways. Exogenous GA activates and promotes nitrogen metabolism (H. Sun et al., 2021), thereby inducing nicotine synthesis. While MT-induced antioxidant responses may interfere with carbon-nitrogen resource allocation (Erdal, 2019), ultimately causing metabolic disruption.

Notably, combined applications at medium concentrations (25–50 mg/L GA_3_ + 40–120 μmol/L MT) exhibited high instability in regulating industrial usability (CCUI, 46.56–58.50). This volatility may stem from the disruption of the balance between tobacco chemical compounds. For example, the extreme enrichment of chloride ion in tobacco leaf leading to an imbalanced K/Cl ratio, and results in combustibility of cured tobacco leaves (Bell et al., 1988).

Our results revealed a nonlinear response pattern of upper leaf industrial usability (CCUI) to GA_3_ and MT co-application. The superior performance of optimal single-hormone treatments indicates that both GA_3_ and MT can improve upper leaf quality through independent pathways. When GA_3_ >50 mg/L combined with MT >80 μmol/L, antagonistic effects dominate metabolic processes, causing dual imbalances in the sugar-to-alkaloid ratio and K-to-Cl ratio. The nonlinear response of chemical composition to GA_3_ and MT co-application indicates that these two hormones regulate tobacco metabolism through partially independent but intersecting pathways. At optimal single-hormone concentrations, GA_3_ mainly modulates nitrogen metabolism and alkaloid synthesis, while MT enhances carbon assimilation and ionic homeostasis. However, excessive co-application may disrupt carbon–nitrogen allocation and ion balance, leading to abnormal alkaloid accumulation and reduced sugar content. Future research is required to investigate the interaction nodes between these two hormones in carbon-nitrogen allocation within the tobacco plant and validation of the stability of single-application strategies (75 mg/L GA_3_ or 80 μmol/L MT) under field conditions. This will provide a theoretical basis and practical application in tobacco production for the targeted regulation of chemical quality in upper tobacco leaves.

It should be emphasized that CCUI represents a comprehensive score derived exclusively from chemical composition indicators and is therefore used to evaluate the industrial usability and chemical quality of tobacco leaves. In contrast, the TOPSIS model integrates CCUI together with leaf area, fresh weight, and dry weight to identify treatments with optimal overall performance. Consequently, a treatment exhibiting the highest CCUI does not necessarily correspond to the best comprehensive treatment when growth-related traits are simultaneously considered.

## 5 Conclusion

Upper tobacco leaves often suffer from typical defects, including insufficient leaf opening (small leaf area), compact leaf structure, and unbalanced chemical composition (e.g., high alkaloids and low sugar–alkaloid ratio), which restrict their industrial utilization. This study demonstrates that GA is the dominant factor alleviating the morphological limitation of upper leaves: spraying 75 mg/L GA_3_ after topping significantly increased upper-leaf width and leaf area, and markedly enhanced fresh and dry biomass accumulation. In addition, GA ×MT interaction significantly improved leaf photosynthetic performance, providing physiological support for biomass formation. Regarding chemical quality, single-hormone treatments at appropriate concentrations improved chemical coordination; 80 μmol/L MT (A1B3) achieved the highest CCUI, whereas excessive co-application caused imbalanced sugar–alkaloid and K/Cl ratios, leading to reduced CCUI. Importantly, because CCUI evaluates chemical usability only, while TOPSIS integrates CCUI with growth-related traits (leaf area, fresh weight, and dry weight), the optimal treatment differs across evaluation targets: MT (A1B3) is preferable for chemical-quality improvement, whereas 75 mg/L GA_3_ + 80 μmol/L MT (A4B3) represents the best overall strategy to simultaneously promote growth and maintain acceptable chemical coordination. These findings provide a practical hormone-based approach to mitigating upper-leaf defects and improving the industrial utilization potential of upper tobacco leaves.

## Data Availability

The original contributions presented in the study are included in the article/supplementary material. Further inquiries can be directed to the corresponding author.
